# Altered expression of epithelial-to-mesenchymal transition proteins in extraprostatic prostate cancer

**DOI:** 10.18632/oncotarget.6689

**Published:** 2015-12-19

**Authors:** Clare Verrill, Lucia Cerundolo, Chad Mckee, Michael White, Christiana Kartsonaki, Eve Fryer, Emma Morris, Simon Brewster, Indrika Ratnayaka, Luke Marsden, Hans Lilja, Ruth Muschel, Xin Lu, Freddie Hamdy, Richard J. Bryant

**Affiliations:** ^1^ Department of Cellular Pathology, Oxford University Hospitals NHS Foundation Trust, John Radcliffe Hospital, Headington, Oxford, UK; ^2^ Nuffield Department of Surgical Sciences, University of Oxford, Headington, Oxford, UK; ^3^ CRUK/MRC Oxford Institute for Radiation Oncology, University of Oxford, Headington, Oxford, UK; ^4^ Ludwig Institute for Cancer Research Ltd, University of Oxford, Nuffield Department of Clinical Medicine, Headington, Oxford, UK; ^5^ Department of Oncology, University of Oxford, Headington, Oxford, UK; ^6^ Department of Urology, Churchill Hospital, Headington, Oxford, UK; ^7^ Departments of Surgery (Urology Service), Laboratory Medicine (Clinical Chemistry Service) and Medicine (Genitourinary Oncology Service), Memorial Sloan Kettering Cancer Center, New York, NY, USA; ^8^ Department of Laboratory Medicine and Clinical Sciences in Malmö, Lund University, Skåne University Hospital, Malmö, Sweden; ^9^ Institute of Biomedical Technology, University of Tampere, Tampere, Finland

**Keywords:** epithelial to mesenchymal transition, extraprostatic prostate cancer, immunohistochemistry, *in vitro* organotypic cell culture, *in vivo* mouse prostate cancer model, Pathology Section

## Abstract

Epithelial to mesenchymal transition (EMT) of cancer cells involves loss of epithelial polarity and adhesiveness, and gain of invasive and migratory mesenchymal behaviours. EMT occurs in prostate cancer (PCa) but it is unknown whether this is in specific areas of primary tumours. We examined whether any of eleven EMT-related proteins have altered expression or subcellular localisation within the extraprostatic extension component of locally advanced PCa compared with other localisations, and whether similar changes may occur in *in vitro* organotypic PCa cell cultures and *in vivo* PCa models. Expression profiles of three proteins (E-cadherin, Snail, and α-smooth muscle actin) were significantly different in extraprostatic extension PCa compared with intra-prostatic tumour, and 18/27 cases had an expression change of at least one of these three proteins. Of the three significantly altered EMT proteins in pT3 samples, one showed similar significantly altered expression patterns in *in vitro* organotypic culture models, and two in *in vivo* Pten^−/−^ model samples. These results suggest that changes in EMT protein expression can be observed in the extraprostatic extension component of locally invasive PCa. The biology of some of these changes in protein expression may be studied in certain *in vitro* and *in vivo* PCa models.

## INTRODUCTION

It is estimated that 220,800 men in the United States will be diagnosed with prostate cancer (PCa) during 2015 and 27,540 men will die from this malignancy [1], usually from metastatic disease. Within the TNM classification of PCa, the pT3a stage of this malignancy describes extraprostatic extension of the tumour beyond the prostatic capsule. Extraprostatic extension of PCa is associated with recurrence and metastasis following radical treatment [2-5]. Adjuvant treatment after radical prostatectomy (RP) depends on histopathological findings predictive of recurrence and survival, such as surgical margin status and pathological stage [6-8]. Biomarkers are associated with clinical outcome following RP, but none are routinely used in clinical practice as they have not demonstrated additional prognostic value and clinical utility beyond standard care.

Epithelial to mesenchymal transition (EMT) describes loss of epithelial cell polarity and adhesion, and gain of migratory invasive mesenchymal behaviours [9]. EMT occurs during embryogenesis, and can promote adenocarcinoma cell invasiveness and metastasis [10, 11]. EMT occurs in several cancers including PCa [12-18] and can predict recurrence after RP [19, 20], but it is unknown whether it occurs in specific areas of primary PCa such as extraprostatic extension in pT3a tumours. We investigated potential differences in EMT protein expression between the extraprostatic extension and intraprostatic tumour components of pT3a PCa in RP specimens. In light of the increasing research interest in the process of EMT as a mechanism of PCa progression, we also investigated whether *in vitro* and *in vivo* PCa models commonly used in the laboratory to investigate mechanisms of PCa cellular invasion such as EMT demonstrate any similar changes to those observed in human pT3a RP specimens.

## RESULTS

**Figure 1:**
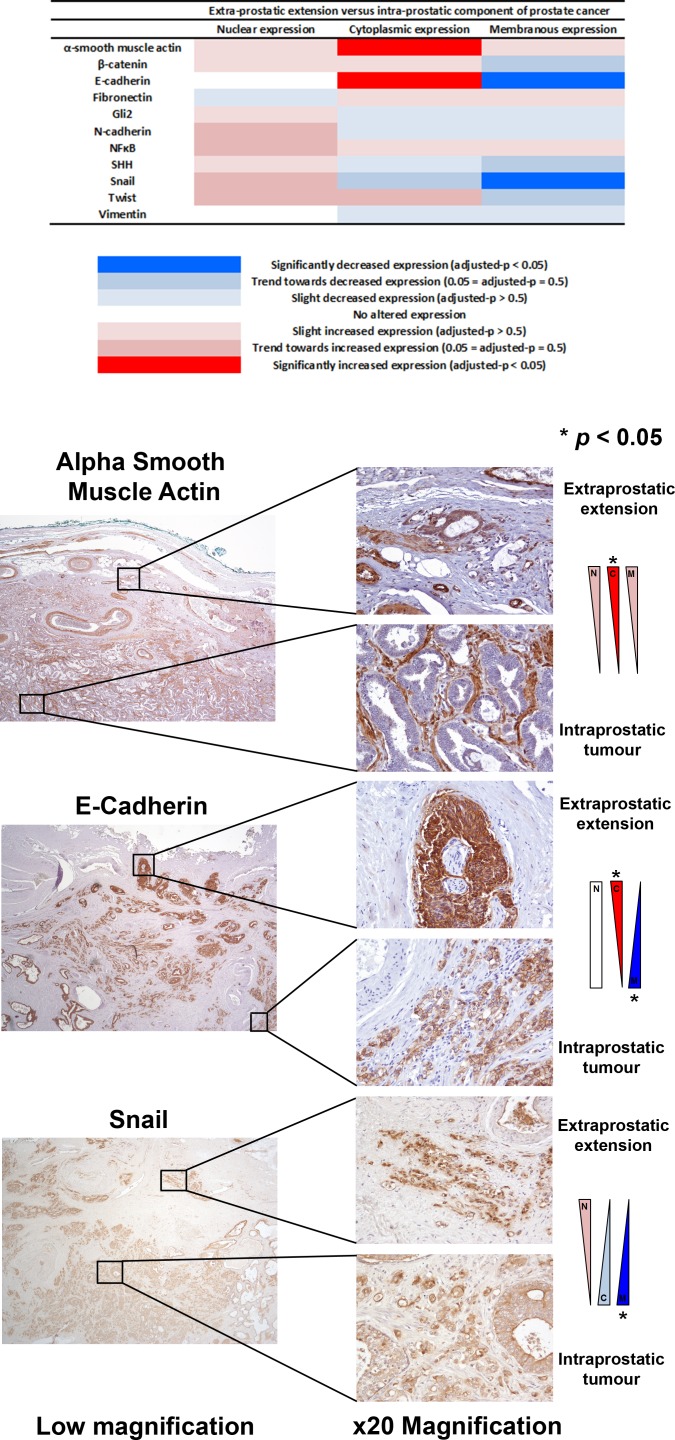
Analysis of EMT-related protein expression changes in locally advanced invasive prostate cancer The expression profiles of eleven candidate EMT-related proteins were analysed in extraprostatic and intraprostatic regions of 27 cases of locally advanced invasive prostate cancer in radical prostatectomy specimens. Significantly increased cytoplasmic α-smooth muscle actin, increased cytoplasmic E-cadherin, decreased membranous E-cadherin, and decreased membranous Snail expression (*adjusted-*p* < 0.05 for each) was observed in extraprostatic foci of prostate cancer compared with intraprostatic tumour.

**Figure 2:**
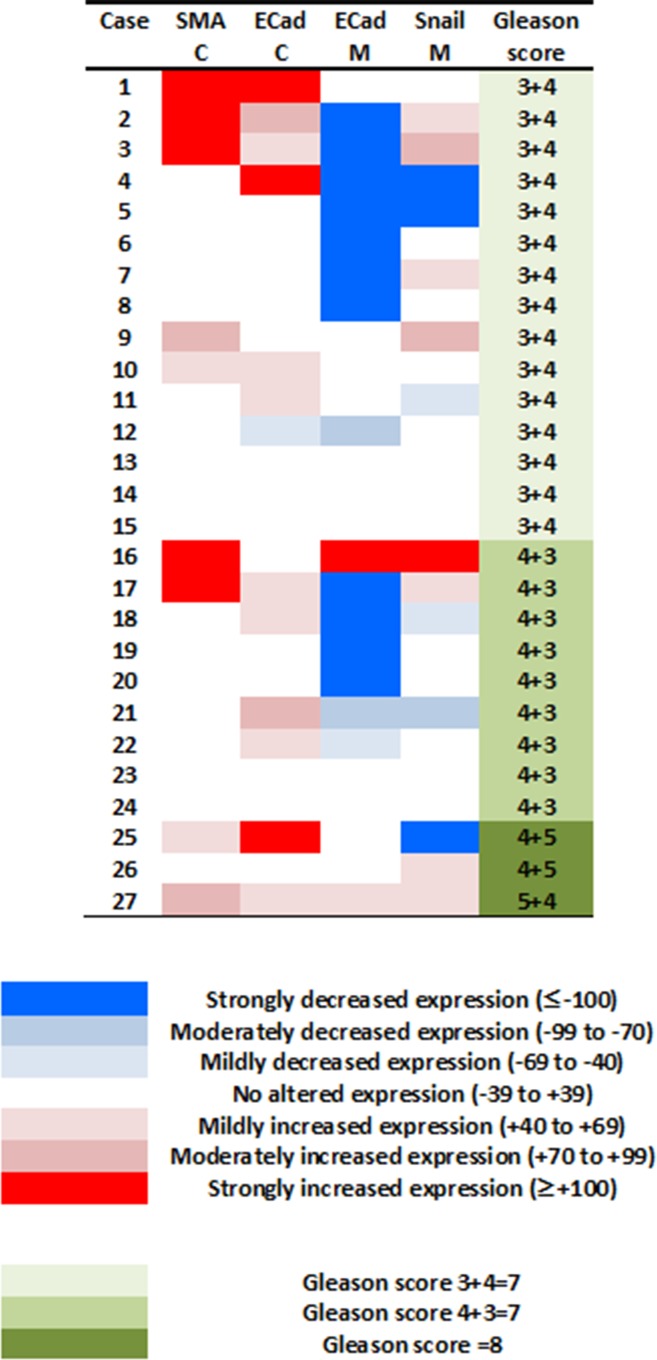
Investigating potential associations between the degree of changes in expression of EMT-related proteins at the extraprostatic component of pT3a prostate cancer, and prostate cancer Gleason grade The 27 cases of pT3a prostate cancer demonstrated variability with regards to their changes in expression of EMT-related proteins (colour key: ≤ −100 strongly, −99 to −70 moderately, and −69 to −40 mildly decreased expression; −39 to +39 no altered expression; +40 to +69 mildly, +70 to +99 moderately, and ≥ +100 strongly increased expression). These cases demonstrated heterogeneous EMT protein expression, with 18/27 (67%) showing moderate or strong expression changes of at least one of these three EMT-related proteins. No significant relationship was observed between the number of EMT-related proteins altered in the extraprostatic versus intraprostatic component of these pT3a tumours and the Gleason sum score (*p* > 0.05) or primary Gleason grade pattern (*p* > 0.05).

**Figure 3:**
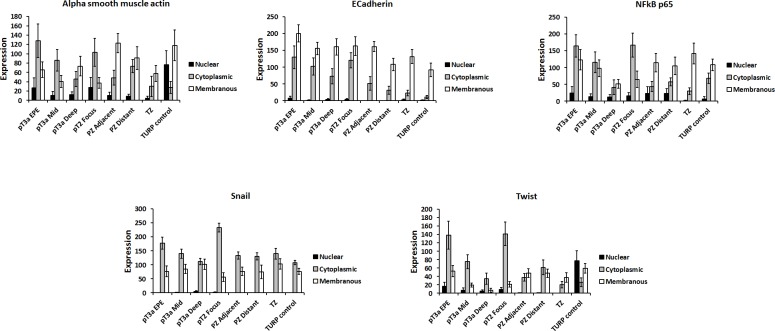
Similar changes in EMT-related protein expression to those observed in whole-mount specimens can be observed within discrete foci of abnormality within a “heterogeneity tissue microarray” This microarray contains 7 cores (one from each of the extraprostatic extension component, central tumour, and “deep” tumour of the “index” prostate cancer, one from a synchronous focus of pT2 prostate cancer, one from each of the adjacent and distant benign peripheral zone, and one from the transitional zone) from each of 13 radical prostatectomy cases. A trend towards increased cytoplasmic α-smooth muscle actin and increased cytoplasmic E-cadherin was observed in extraprostatic extension versus pT3a “mid-” and “deep”-areas in these samples. For several EMT-related proteins the pattern of expression in the extraprostatic extension component of pT3a disease was similar to that observed in a separate focus of pT2 disease.

The expression of EMT proteins in whole mount sections from 27 cases of pT3a PCa (Table 1) was analysed. We observed significantly altered expression of three proteins in extraprostatic extension versus intraprostatic cancer (increased cytoplasmic α-smooth muscle actin, decreased membranous and increased cytoplasmic E-cadherin, and decreased membranous Snail; adjusted-*p* < 0.05 for each) (Figure 1). These cases demonstrated heterogeneous EMT protein expression, with 18/27 (67%) showing “strong” or “moderate” expression changes of at least one of these three EMT-related proteins (Figure 2). The most consistent alteration in EMT-related protein expression in these clinical cases is seen for membranous E-cadherin, with 11 of the 27 pT3a cases demonstrating strong decreased expression in extraprostatic extension versus intraprostatic tumour. No statistically significant association was seen between EMT-related protein expression changes in the extraprostatic extension versus intraprostatic component of pT3a disease and Gleason sum score of the pT3a focus of tumour. Next, we looked to see if any of the changes in EMT-related protein expression observed in the pT3a wholemount sections could be seen in cases from a “heterogeneity” tissue microarray comprising samples from 13 patients and including tissue cores from the extraprostatic extension focus, “mid-portion”, and “deep” focus of pT3a PC, a separate pT2 focus, and from various histologically benign regions of the prostate (Table 2). A trend towards increased cytoplasmic α-smooth muscle actin and increased cytoplasmic E-cadherin was observed in extraprostatic extension versus pT3a “mid-” and “deep”-areas (Figure 3) in these TMA samples. For several EMT-related proteins the pattern of expression in the extraprostatic extension component of pT3a disease was similar to that observed in a separate focus of pT2 disease (Figure 3), despite the fact that the Gleason scores of the extraprostatic extension component of the pT3a samples in the “heterogeneity” TMA were significantly different from those of the separate pT2 foci (Table 2).

**Table 1:** Demographics and baseline radical prostatectomy pT3a prostate cancer characteristics

	pT3a prostate cancer (*n* = 27)
*Median age* (range)	63 (48-73) years
	
*Preoperative PSA*	
≤10	21
10.1-20	4
>20	2
	
*pN-stage*	
pNX pN0	9 17
≥pN1	1
	
*Gleason grade*	
≤6	0
7 (3+4)	15
7 (4+3)	9
≥8	3

**Table 2:** Analysis of Gleason grades of separate prostate cancer foci within a “heterogeneity” prostate cancer tissue microarray

TMA Case	EPE focus of pT3a tumour	Main pT3a tumour	Separate pT2 focus of tumour
1	3+4	3+3	3+3
2	3+4	3+4	3+4
3	4+3	4+3	3+4
4	3+4	3+4	3+3
5	Absent	3+4	3+4
6	4+3	4+3	3+3
7	4+3	4+3	3+4
8	4+4	4+4	3+4
9	3+4	3+4	4+3
10	3+4	3+4	3+4
11	3+4	4+3	3+3
12	3+4	3+4	3+3
13	4+3	3+4	3+4

	**Gleason score 3+3=6**
	**Gleason score 3+4=7**
	**Gleason score 4+3=7**
	**Gleason score 4+4=8**

Gleason sum score comparisons	p-value
pT3a EPE versus separate pT2 focus	0.005
pT3 main focus versus separate pT2 focus	0.019
pT3a EPE versus main pT3 focus	0.69

The “heterogeneity” tissue microarray comprises samples from 13 patients and includes tissue cores from the extraprostatic extension focus, “mid-portion”, and “deep” focus of pT3a prostate cancer, a separate pT2 focus, and from various histologically benign regions of the prostate. The Gleason scores of the extraprostatic extension component of the pT3a samples in the “heterogeneity” tissue microarray were significantly different from those of the separate pT2 foci.

**Figure 4:**
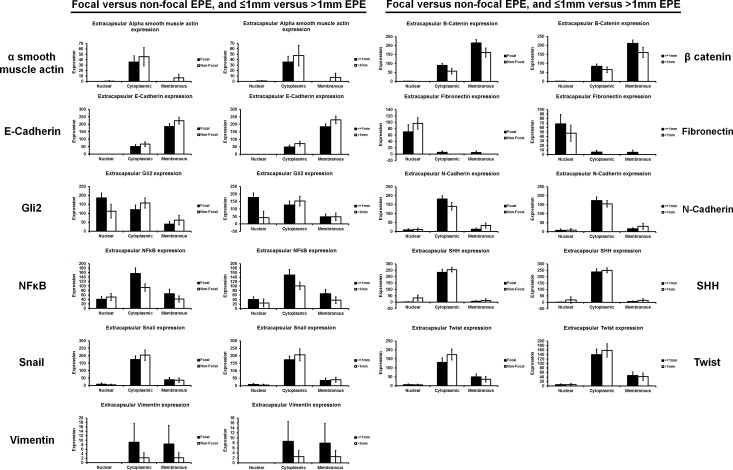
The expression of EMT-related proteins was unrelated to the degree of prostate cancer extraprostatic extension Extraprostatic extension can be classified as focal and non-focal, or ≤1mm and >1mm [2]. No significant difference in the expression of EMT-related proteins was seen between either type of extraprostatic extension classification.

No significant differences in EMT-related protein expression in the x27 wholemount pT3a were observed between various subclassifications of extraprostatic extension [2] (Figure 4). Control experiments using the pT3a wholemount samples excluded immunohistochemistry (IHC) “edge artefacts” and tissue trauma (Figure S1). In order to analyse interobserver reproducibility two uropathologists independently scored α-smooth muscle actin (αSMA) and E-cadherin expression in 10 randomly selected cases of pT3 PCa. Moderate agreement was observed between independent uropathologists for αSMA (κ score 0.438) and fair agreement was observed for E-cadherin (κ score 0.213).

**Figure 5:**
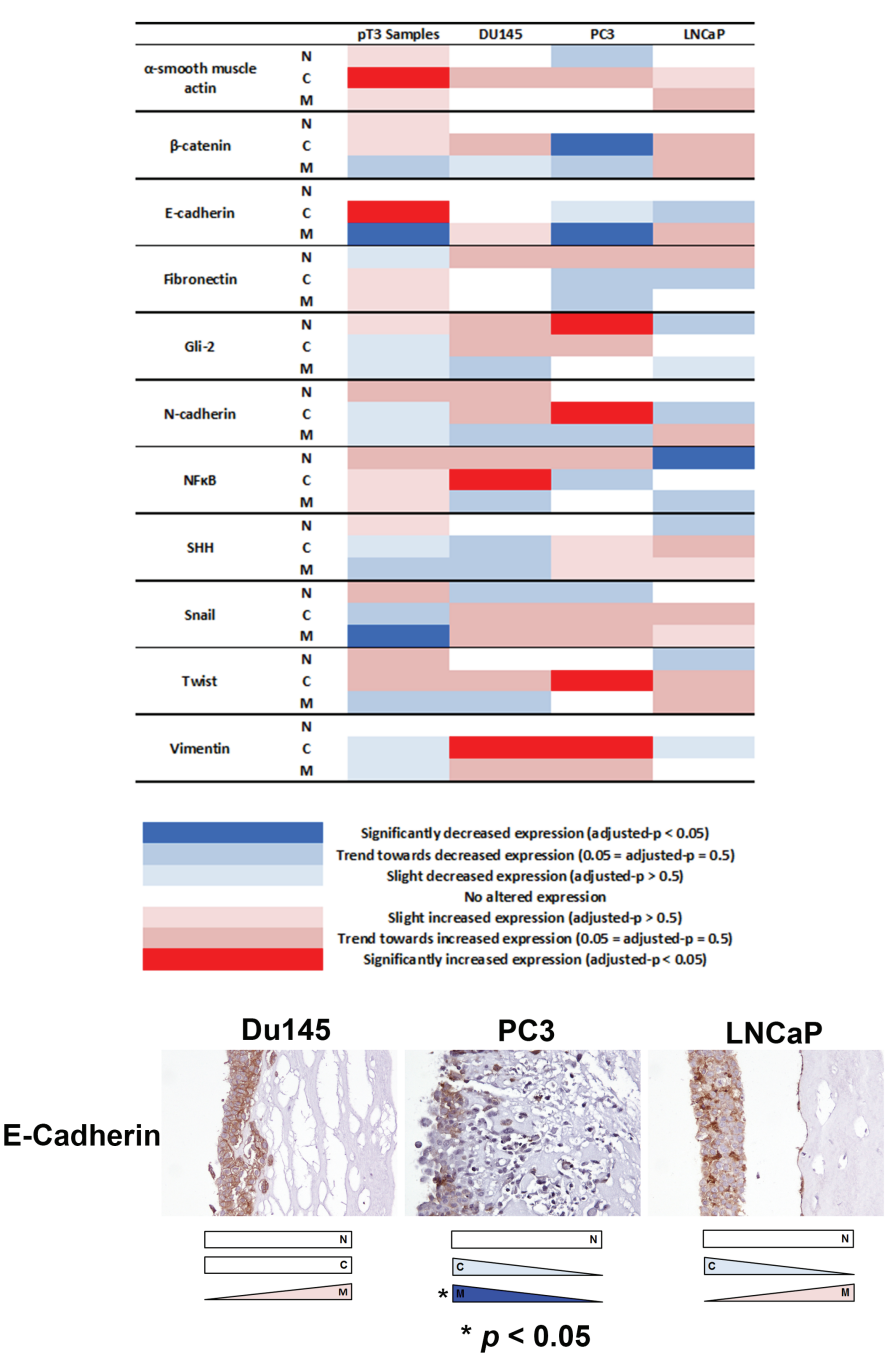
Analysis of EMT-related protein expression in an *in vivo* organotypic culture model of cellular invasiveness Of the four changes in EMT-related protein expression seen in human pT3 samples, decreased membranous E-cadherin expression was also observed in invasive PC3 prostate cancer cells in an *in vitro* organotypic culture model of cellular invasiveness (*p* < 0.05). Several other significant changes in EMT-related protein expression were seen in PC3, DU145 and LNCaP cells in this *in vitro* organotypic cell culture model. N - nuclear, C - cytoplasmic, M - membranous.

Next, we investigated whether EMT protein expression changes observed in extraprostatic extension samples might occur in *in vitro* organotypic cultures of invasive PCa cells. A similar expression change of membranous E-cadherin was observed in invading PC3 cells compared with non-invading upper surface cells (*p* < 0.05) (Figure 5). We also observed decreased cytoplasmic β-catenin (PC3), increased nuclear Gli2 (PC3), increased cytoplasmic N-cadherin (PC3), decreased nuclear NFκB p65 (LNCaP), increased cytoplasmic NFκB p65 (DU145), increased cytoplasmic Twist (PC3) and increased cytoplasmic vimentin (PC3 & DU145) expression (*p* < 0.05 for each) in the *in vitro* organotypic culture cell line experiments.

**Figure 6:**
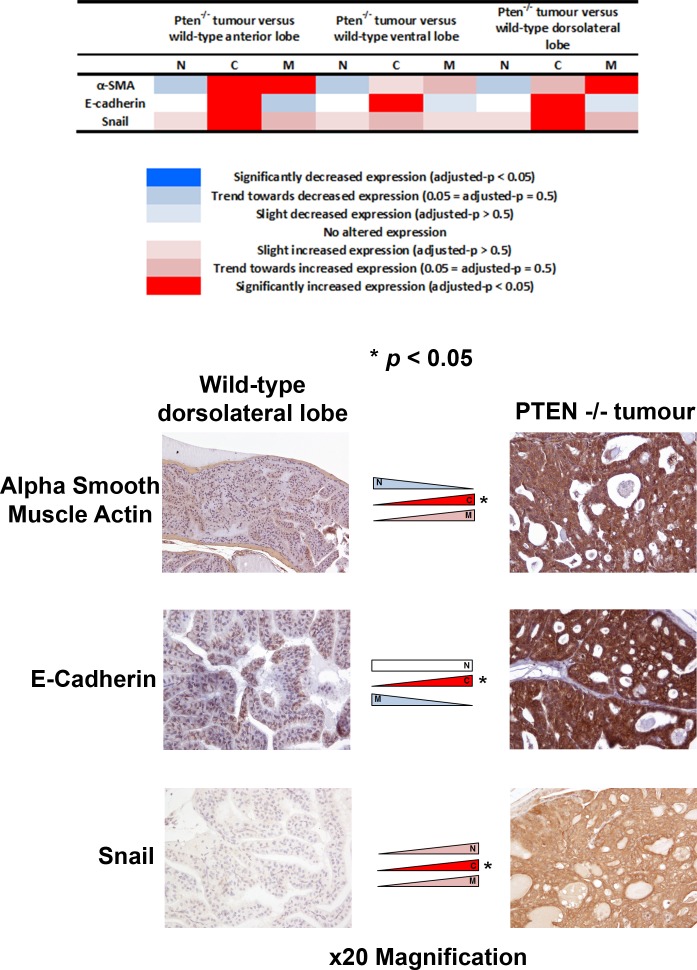
Analysis of EMT-related protein expression in a Pten^−/−^ *in vivo* model of invasive prostate cancer The expression profiles of the three significantly altered EMT-related proteins in human pT3 prostate cancers were studied in an *in vivo* Pten^−/−^ model of invasive prostate cancer. Two of the EMT-related proteins that were altered in human samples (increased cytoplasmic alpha smooth muscle actin and E-cadherin) showed similar changes in expression in prostate cancer cells in the Pten^−/−^ model compared with benign prostate epithelial cells in Pten^+/+^ mice (adjusted-*p* < 0.05).

Finally, we investigated whether EMT expression changes might occur in an *in vivo* Pten^−/−^ mouse model of invasive PCa. Significant expression changes of α-smooth muscle actin, E-cadherin and Snail were observed in tumours in Pten^−/−^ mice (*n* = 7) compared with benign Pten^+/+^ mice (*n* = 6) (adjusted-*p* < 0.05) (Figure 6).

## DISCUSSION

These results suggest EMT occurs in extraprostatic extension components of locally advanced PCa. The mechanisms driving PCa cell metastasis are unclear, and it is unknown whether metastatic clones originate within the extraprostatic extension component or from clones within intraprostatic tumour, although a recent study suggests the latter is possible [21]. Several models of PCa cellular invasiveness exist [22], including cohesive groups of cells budding away from precursor lesions [23], and single cancer cells undergoing EMT and invading stroma and vasculature [24, 25]. Our study builds on existing evidence [18] by demonstrating that EMT occurs in PCa, and when observed in other cancers EMT contributes to metastasis [26]. EMT in cancer invasion is not universally observed [27-30], and distinguishing cancer cells with a mesenchymal phenotype from stromal cells or fibroblasts can be difficult. Our results suggest changes in EMT-related protein expression in the extraprostatic extension component of PCa is not an “all or nothing” phenomenon, and co-expression of mesenchymal markers in EMT in PCa is reportedly uncommon [18].

PCa is often multi-focal with intra-tumoral genomic heterogeneity, and EMT may be heterogeneous. Several of the protein expression changes we observed in extraprostatic extension have been reported elsewhere, including PCa, such as reduced membranous [12] and increased cytoplasmic E-cadherin [31-34], and increased cytoplasmic α-smooth muscle actin [35]. Snail is a transcription factor overexpressed during PCa EMT [36], therefore the reduced membranous Snail observed in extraprostatic extension in our study, and the increased cytoplasmic Snail seen in the Pten^−/−^ mouse model, along with the difference in subcellular localisation of Snail between human, cell line and mouse samples, is difficult to explain, but may represent altered subcellular localisation of a multi-functional protein in these different sample types. Our observation that vimentin was expressed in some benign prostate epithelial cells, but not within cancer cells, is intriguing as vimentin is reportedly overexpressed in invasive PCa cells undergoing EMT [37]. However, vimentin expression has been observed in benign prostate epithelium cases with concomitant PCa [18, 38]. Heterogeneous expression of vimentin in different intraprostatic locations might account for these differences in subcellular localisation. Whilst studies have shown that EMT correlates with adverse features in malignancies including PCa [39-47], we did not observe significant associations between the degree of protein expression change and Gleason score, possibly due to sample size.

The observations in “heterogeneity” TMA experiments suggesting that, for at least some of the EMT-related protein expression changes, the separate pT2 focus of tumour looked similar to the pT3a extraprostatic extension component, were intriguing. These results cannot be easily explained, and were not due to differences in the relative Gleason sum scores of these separate tumour foci. It is an intriguing possibility that metastases may arise from seeding foci within areas of pT2 PCa, rather than from PCa cell clones within the extraprostatic extension region of pT3a foci, and this warrants further investigation. Indeed, it is evident that metastatic clones of cells may arise from intraductal PCa [21]. Moreover, EMT-related changes are not an “all or nothing” phenomenon, therefore some of these changes may be seen in separate pT3a and pT2 tumour foci in multi-focal PCa. This requires further investigation.

The observation that several EMT-related proteins with differential expression in extraprostatic versus intraprostatic cancer were similarly altered in a Pten^−/−^ *in vivo* model of PCa suggests these changes may be biologically relevant in these tumours. Significant changes in expression of EMT proteins were seen in the PCa cells (PC3 and DU145) in *in vitro* organotypic cultures demonstrating “collective cell invasion” [48]. The fact that only PC3 cells demonstrated any similar changes to the observations in RP tumours suggests that some aspects of human PCa cell biology cannot be fully recapitulated by *in vitro* cell line experiments. Selection of cell lines for particular experiments will depend on aspects of cell biology under investigation.

A limitation of our study is the sole focus on IHC changes in EMT-related proteins within extraprostatic extension versus intraprostatic tumour, which are recognized to be potentially variable. Whilst IHC is the commonest current tool in diagnostic pathology, it is important for future studies to investigate potential changes in PCa biology at the extraprostatic extension foci of pT3a tumours using molecular biology techniques such as sequencing. It would be beneficial for future studies to validate the sub-cellular location of these EMT-related proteins using sub-cellular samples extracted from freshly acquired tissues.

A further limitation of this study is the difficulty assessing distinct areas of extraprostatic extension in Pten^−/−^ *in vivo* mouse models of invasive PCa, and then comparing this with intraprostatic tumour, as the mouse prostate lacks a discrete capsule [49]. Nevertheless, we observed significantly increased cytoplasmic aSMA and E-cadherin expression in both extraprostatic extension locally invasive advanced human PCa samples and malignant cells within the *in vivo* mouse model, suggesting that it might be worthwhile investigating the significance of changes in subcellular localization of these EMT-related proteins in further studies.

It will be important to determine whether pT3a PCa with extraprostatic extension EMT has different outcomes following radical surgery compared with matched tumours without EMT. This may influence decision-making regarding post-operative salvage radiotherapy. A larger study with longer clinical follow-up is required to investigate this. Experiments to identify potential clinical utility of analysis of EMT-related protein expression in prostate biopsies are warranted.

In summary our results suggest that changes in expression of EMT-related proteins occur in the extraprostatic extension component of locally invasive PCa, and these changes can be observed in *in vitro* and *in vivo* PCa models commonly used in the laboratory to studies mechanisms of PCa cellular invasion.

## MATERIALS AND METHODS

### Radical prostatectomy specimens

Whole mount sections from formalin-fixed paraffin-embedded pT3a PCa samples from 27 patients treated with RP were reviewed by a uropathologist to identify extraprostatic extension on diagnostic H&E sections. 4μm sections were cut from mega paraffin blocks containing 4mm transverse slices of prostate tissue. The area with extraprostatic extension was selected on the water bath after cutting to ensure that it was included on each whole mount section. This study had appropriate ethical approval (ProMPT ethics reference number MREC 01/4/61, P.I. Professor FC Hamdy).

### Tissue microarray specimens

The “heterogeneity” TMA contains 7 cores (one from each of the extraprostatic extension, central tumour, and “deep” tumour of the index PCa, one from a synchronous focus of pT2 PCa, one from each of the adjacent and distant benign peripheral zone, and one from the transitional zone) from each of 13 RP cases. The construction of the “heterogeneity” TMA had appropriate ethical approval (Oxford Radcliffe Biobank ethics reference number 09 H0606 5+5).

### Organotypic cultures

Organotypic cultures of PC3, DU145 and LNCaP cells were generated as described previously [50-53].

### Pten^−/−^ mouse prostate cancer samples

Prostate samples from each prostate lobe (ventral, anterior and dorsolateral) from 6 Pten^+/+^, and from each invasive PCa from 7 Pten^−/−^ mice, were analysed.

### Antibodies

Primary antibodies (Abcam, UK, and 1:50 dilution unless otherwise stated) used for IHC included rabbit anti-α smooth muscle actin (ab5694, 1:200), mouse anti-β-catenin (M3539, DAKO Ltd, UK, 1:100), rabbit anti-E-cadherin (ab53033, 1:1500), rabbit anti-Fibronectin (ab2413, 1:150), rabbit anti-Gli2 (ab26056), rabbit anti-N-cadherin (ab12221, 1:400), rabbit anti-NFκB p65 (ab7970, 1:1000), rabbit anti-Sonic Hedgehog (ab53281), goat anti-Snail (AF3639, R&D Systems, USA), rabbit anti-Twist (ab49254, 1:100), mouse anti-Vimentin (clone V9, M0725, DAKO Ltd, UK, 1:100). Secondary antibodies used included goat biotinylated anti-mouse IgG (BA-9200, Vector Laboratories, USA, 1:250), goat biotinylated anti-rabbit IgG (BA-1000, Vector Laboratories, USA, 1:250), and ImmPRESS peroxidase reagent kit (antigoat MP-7405 and anti-rabbit MP-7401, Vector Laboratories, USA).

### Immunohistochemistry and immunocytochemistry

IHC was performed as previously described [50] with boiling sodium citrate buffer pH 6 antigen retrieval for α-smooth muscle actin, β-Catenin, E-cadherin, Fibronectin, Gli2, N-cadherin, NFκB p65, Sonic Hedgehog, Twist and Vimentin. All immunohistochemistry experiments included negative controls by omitting the primary antibody.

### Semi-quantitative protein expression analysis

Nuclear, cytoplasmic and membranous localisation of each EMT-related protein in luminal prostate epithelial cells in extraprostatic extension and intraprostatic (defined as the “deepest” area of the same tumour away from the capsule) PCa, and histologically benign epithelium, in a x4 field of whole mount RP specimens and in “heterogeneity” TMA sections was quantified by a uropathologist. For each protein an intensity (0-3) and cell percentage was assigned for nuclear, cytoplasmic and membranous expression in PCa cells, and these were multiplied to give an expression score (range 0-300) for each subcellular compartment. Features of extraprostatic extension including focality, radial distance and location were noted. The expression of each protein was similarly quantified for PCa cells situated within the invasive margin or non-invasive upper layer of *in vitro* organotypic cultures, and within the invasive margin and central tumour in *in vivo* Pten^−/−^ mouse PCa.

“Control” analyses were performed for RP experiments to exclude histological artefacts. To exclude differential fixation in the peripheral and central prostate regions, and to exclude “edge artefact’’ (where the section edge lifts creating darker staining), SHH and E-cadherin expression was quantified at both the extraprostatic extension component of pT3a PCa and the innermost “cut edge” of the tumour sample in a random whole mount section, and the paired samples were compared.

We undertook an analysis of interobserver reproducibility using 10 randomly selected cases from the samples stained with anti-αSMA and E-cadherin antibodies. Reproducibility of protein expression scoring for the re-reviewed cases was assessed using κ statistics. By convention, a κ score <0 is poor interobserver agreement, 0.0-0.20 slight agreement, 0.21-0.40 fair agreement, 0.41-0.60 moderate agreement, 0.61-0.8 substantial agreement and >0.80 almost perfect agreement [54].

### Statistical analysis

Human pT3 (*n* = 27) extraprostatic and intraprostatic EMT-related protein expression was compared using a linear regression model with log-transformed expression scores as the response and extra-/intra-prostatic as an explanatory variable. This was performed separately for each EMT-related protein and for each of the three cellular compartments (nuclear, cytoplasmic and membranous). *p* values were adjusted for multiple testing using the Benjamini-Hochberg procedure and additionally they were compared to their expected values.

EMT-related protein expression in Pten^−/−^ mice (*n* = 7) was compared with expression in each of the three lobes (dorsolateral, anterior and ventral) containing benign prostate epithelium in wild-type Pten^+/+^ mice (*n* = 6) using a linear regression model with log-transformed expression scores as the response. This was performed separately for each EMT-related protein and for each of the three cellular compartments (nuclear, cytoplasmic and membranous), adjusting for multiple testing as for human pT3 samples above.

EMT-related protein expression in cell lines was analysed by paired t-tests comparing log-transformed values for the innermost layer (invading) and outermost layer (non-invading) of cells for each cell line and each cellular compartment (nuclear, cytoplasmic and membranous). The results were not adjusted for multiple testing because *n* = 3 for each cell line and each cellular compartment.

To assess any potential relationship between the presence of an EMT-related protein expression difference between extraprostatic and intraprostatic human PCa, and primary Gleason pattern being ≥4, logistic regression was used with the difference in extraprostatic and intraprostatic protein expression as an explanatory variable, both with and without adjustment for intraprostatic expression. The effect of the difference between the extraprostatic and intraprostatic component on recurrence was also examined using logistic regression.

## SUPPLEMENTARY MATERIAL FIGURE


