# Supplementary material for: Altered expression of epithelial-to-mesenchymal transition proteins in extraprostatic prostate cancer
Source: Oncotarget. 2015 Dec 19;7(2):1107–19. doi: 10.18632/oncotarget.6689 (PMC4811447; doi:10.18632/oncotarget.6689)
Supplement: Supplementary file 1 [file oncotarget-07-1107-s001.pdf]

Altered expression of epithelial-to-mesenchymal transition proteins in extraprostatic prostate cancer

Supplementary Material

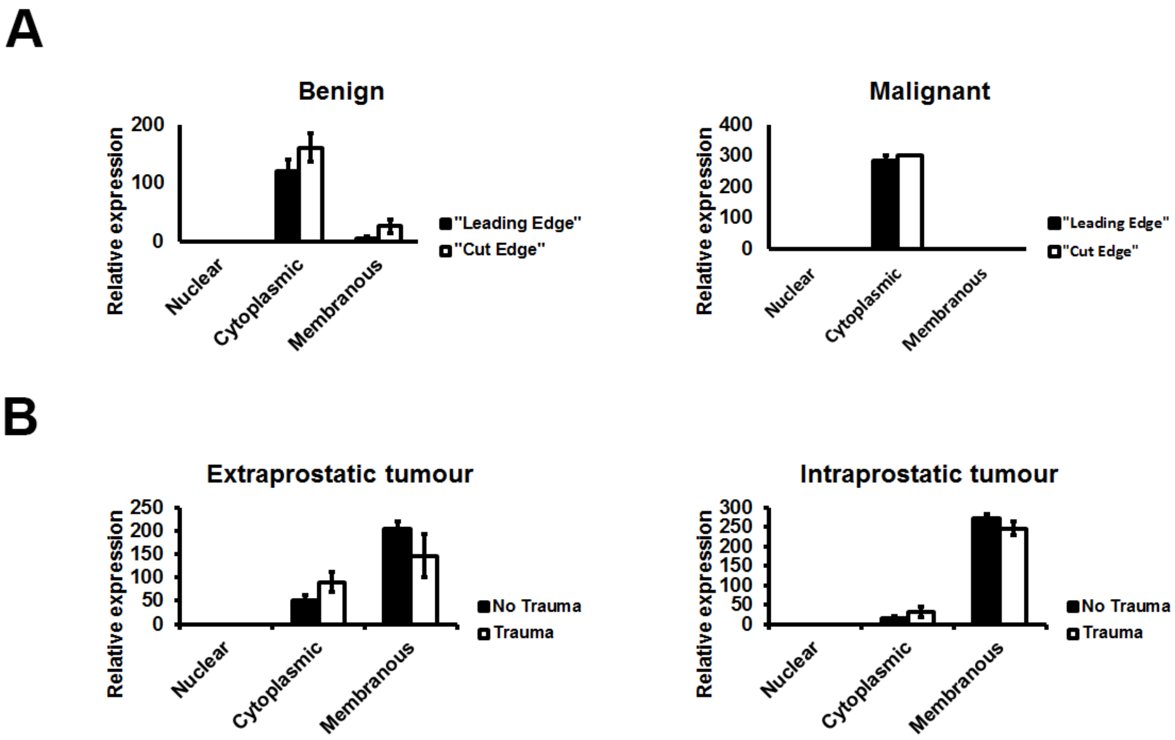

Figure S1. Control experiments excluded immunohistochemistry "edge artefacts" and "tissue trauma artefacts". (A) No significant difference was observed between the expression of SHH in the extraprostatic component of prostate cancer in the whole mount specimen compared with the inner "cut edge" of the tumour sample, thereby excluding the possibility of an "edge effect" of the immunostaining. **(B)** No significant difference was observed in the expression of E-cadherin in the extraprostatic extension component of pT3a prostate cancer samples when the presence or absence of significant tissue trauma to the specimen was accounted for.
